# Roles of GFPT2 Expression Levels on the Prognosis and Tumor Microenvironment of Colon Cancer

**DOI:** 10.3389/fonc.2022.811559

**Published:** 2022-03-07

**Authors:** Xiaorong Ding, Hua Liu, Ying Yuan, Qin Zhong, Xiaomin Zhong

**Affiliations:** Department of Oncology, The Affiliated Huaian No.1 People’s Hospital of Nanjing Medical University, Huai’an, China

**Keywords:** colon cancer, GFPT2, prognosis, tumor microenvironment (TME), drug sensitivity

## Abstract

**Background:**

Recently, increasing evidence has suggested that Glutamine-fructose-6-phosphate transaminase 2 (GFPT2) is related to carcinogenesis. However, the potential roles of GFPT2 in colon cancer still need to be fully investigated.

**Methods:**

We examined the protein levels of GFPT2 by immunohistochemistry (IHC) in tissues collected from 83 patients with colon cancer. We further detected GFBPT2 protein levels by Western Blot assay. We checked the relationship between GFPT2 expression levels and overall survival (OS), stromal and immune scores and immune components from The Cancer Gene Atlas (TCGA) database. GFBP2-related pathways were validated in the Cancer Cell Line Encyclopedia (CCLE) database. Expression of GFPT2 in single cell subpopulations was calculated from The Tumor Immune Single Cell Center (TISCH). The levels of GFPT2 and drug sensitivity data were performed from CellMiner dataset.

**Results:**

GFPT2 was highly expressed and correlated with poor pathological features in 83 colon cancer patients. Moreover, increased GFPT2 expression was significantly associated with poorer OS in 329 colon adenocarcinoma (COAD) patients. Gene ontology (GO) and Kyoto Encyclopedia of Genes and Genomes (KEGG) analysis showed the differentially expressed genes of GFPT2 were mostly enriched in focal adhesion, ECM receptor interaction, JAK/STAT signaling pathway and immune related pathways. In addition, GFPT2 expression was correlated with the tumor microenvironment (TME). GFPT2 expression was linked to cancer-associated fibroblasts (CAFs)-associated factors and epithelial-mesenchymal transition (EMT)-related factors. GFPT2 was positively correlated with immunosuppressive cells and regulated immunosuppressive factors and T-cell exhaustion. Finally, our data suggested that the expression of GFPT2 may be a judgment of the sensitivity of a certain class of drugs.

**Conclusions:**

Our work reveals the roles of GFPT2 in tumorigenesis, particularly in immune response, TME and drug resistance, which are crucial for the development of customized cancer therapies.

## Background

Colon cancer is a common malignant tumor of the gastrointestinal tract occurring at the junction of the rectum and sigmoid colon, with the highest incidence in the age group of 40 to 50 years old, and the ratio of men to women is 2 to 3:1 (1). Globally, colon cancer is the third and second most commonly diagnosed cancer in men and women, respectively, with 1.9 million new cases and nearly 935,000 deaths in 2020 (2). Incidence and mortality rates are significantly higher in men than in women. Tumor cells in colon cancer usually invade lymphatic vessels, blood vessels or other channels from the primary site and continue to grow there, forming the same type of tumor as the primary site. Metastasis is a characteristic of colon cancer deterioration. Distant metastases from colon cancer are mainly in the liver, and about 50% of patients will have preoperative or postoperative liver metastases. About 30% of patients have insidious liver metastases that cannot be detected by ultrasound or CT before surgery. However, only a small percentage (10%-20%) are suitable for surgical resection, and 70% of them recur postoperatively (3, 4). Therefore, it is crucial to find new treatments for colon cancer.

Tumor cells live in a stressful environment with various important nutrients such as glucose, glutamine and oxygen in dynamic changes (5, 6). Therefore, their major biomolecules including polysaccharides, proteins, lipids, nucleic acid synthesis, and energy and NADPH production are altered to adapt to their survival and proliferation requirements (7). Otto-Warburg first observed in their experiments that tumor tissues can take up large amounts of glucose in the presence of adequate glucose and oxygen levels in the *in vitro* culture environment. Interestingly, however, pyruvate produced by tumor cells through glycolysis was not coupled to the tricarboxylic acid cycle (TCA cycle) in the mitochondria, but was converted to lactate and they called this phenomenon the ‘Warburg effect’ (8). Glutamine-fructose-6-phosphate transaminase (GFPT) is the rate-limiting enzyme of the hexosamine biosynthetic pathway (HBP) and consists of the unlinked highly homologous genes GFPT1 and GFPT2 encoding the transaminase, one of the most common and important pathways in glucose metabolism responsible for glycosylation (9, 10). GFPTs catalyze the formation of glucosamine-6-phosphate from glucosamine and fructose-6-phosphate, and glucosamine infusion can bypass this pathway (11). Recent studies have shown that aberrant expression of GFPTs can lead to reprogramming of fibroblast metabolism in lung adenocarcinoma (12–14) and also GFPT2 is highly expressed in more aggressive breast cancer cell lines (11).

In this study, we found that GFPT2 was highly expressed in colon adenocarcinoma and that high expression was associated with poor pathological features and poor clinical prognosis. By analyzing the signaling pathways of the gene, we found that the gene mediates pathways related to tumor development, and more interestingly, the gene was associated with the tumor microenvironment and immune-related signaling pathways. Overall, our findings uncovered new evidence of functional properties associated with metabolic reprogramming in colon cancer, as well as potential new therapeutic pathways.

## Methods

### Patients and Tissue Samples

We obtained tissue samples from 83 colon cancer patients collected from the Affiliated Huaian No.1 People’s Hospital of Nanjing Medical University. These tissue samples were collected from 2015 to 2021, and they all had complete clinicopathological data, and all samples were de-identified. These data included sex, age, cellular differentiation, TNM stage, primary tumor size, and lymph node metastasis status. All procedures in this study involving human material were approved by the committee of the Affiliated Huaian No.1 People’s Hospital of Nanjing Medical University, and consent forms were acquired from all patients.

### Immunohistochemistry (IHC)

The colon cancer tissues were embedded in paraffin and cut off in sections (4 μm). The tissue samples were de-paraffined with xylene and hydrated with graded ethanol. Slices were treated with 1× citrate repair solution at 100°C for 30 minutes. Next, tissue sections were placed in 3% hydrogen peroxide for 15 minutes at room temperature. Then, the sections were blocked with 5% bovine serum albumin (BSA) (Sigma, A3294) for 1 hour at room temperature. The tissue sections were incubated overnight at 4°C with primary antibody (anti-GFPT2, Thermo Fisher Scientific, PA5-26290, 1:100 dilution). The next day, the sections were incubated with secondary antibody (Goat anti-Rabbit, Dako, P0448) at 37°C for 1 hour. Then DAB (DAB and Substrate Chromogen System, Dako) and hematoxylin staining of the nuclei were performed. The staining intensity was graded as follows: negative- (<10% positive cells), positive+ (11%-30% positive cells); positive++ (31%-50% positive cells); positive+++ (51%-100% positive cells). Negative- and positive+ were considered as low GFPT2 expression, and positive++ and positive+++ were considered as high GFPT2 expression.

### Western Blot

Tissues were cut and digested by RIPA buffer (Beyotime Biotechnology, Shanghai, China) and the concentration of proteins were measured using BCA™ Protein assay kit (ThermoFisher Scientific). Total 30 μg protein was separated by SDS PAGE gels and transferred to 0.2 µm PVDF membrane (Millipore). After blocking with 5% fat free milk, membrane was incubated with primary antibodies (anti-GFPT2, Thermo Fisher Scientific, PA5-26290, 1:1,000 dilution; anti-GAPDH, abcam, ab8245, 1:5,000 dilution) at 4°C overnight. The next day, the membrane was incubated with secondary antibodies (Cell Signaling Technology, #14708 or #14709, 1:10,000 dilution) for 2 hrs at room temperature. Then the membrane was exposed after using ECL reagent Supersignal West Pico chemiluminescent Substrate (ThermoFisher Scientific).

### Data Acquisition

The RNAseq data of COAD samples were downloaded from The Cancer Gene Atlas TCGA dataset (https://portal.gdc.cancer.gov/). The RNAseq data were log-transformed in Fregments Per Kilobase per Million (FPKM) format. Then, the data of normal tissue was removed, and gene expression data and clinical information were merged.

### Survival Analysis

For survival analysis, age was incorporated to exclude the effect of age on survival time, and survival data were statistically analyzed by the survivor R package and visualized visualized for 329 COAD samples by the survminer R package. P value was calculated from Log-rank test.

### Gene Ontology (GO) and Kyoto Encyclopedia of Genes and Genomes (KEGG) Analysis

COAD samples were divided into GFPT2 low and high expression groups for differential analysis, and differentially expressed genes were obtained (P < 0.05). GO and KEGG enrichment analysis was performed using the ClusterProfilter R package, and significant enrichment pathways were obtained (P < 0.05). P values were adjusted using the BH method.

### Validation of the Cancer Cell Line Encyclopedia (CCLE) Database

The CCLE database (https://sites.broadinstitute.org/ccle/) provides high-throughput sequencing results of cell lines from over 1100 different tumor types, including LOVO, HCT116, SW480 and SW620, and a total of 56 intestinal cancer cell lines (15). The genes associated with GFPT2 were analyzed by Pearson using the CCLE database, and the final genes were screened by Pearson correlation coefficient r>0.5 and p-value <0.001.

### Analysis of Stromal and Immune Cell Infiltration

Stromal, immune cell, ESTIMATE and tumor purity scores were obtained by the ESTIMATE (Estimation of STromal and Immune in MAlignant Tumors Using Expression Data) algorithm (16). The stromal, immune scores were used to check the infiltrative expression of stromal and immune cells in COAD. The contents of immune cells in each sample were calculated using CIBERSORT. The correlation coefficient between gene expression and immune cells was calculated by Cor.test function, and the correlation between gene expression and immune cells was tested using spearman. Visualization was performed using the ggpubr R package.

### Single Cell Analysis

The Tumor Immune Single Cell Center (TISCH) (http://tisch.comp-genomics.org/) was used to study the expression of the GFPT2 gene in the tumor microenvironment as a single cell subset. TISCH is a scRNA-seq database focused on the tumor microenvironment (TME). TISCH provides detailed at the single cell level annotation of cell types, allowing one to explore TME in different cancer types (17). In this dataset, there are three main cell types, including immune cells, stromal cells, and malignant cells.

### GFPT2 Co-Expression Heatmap Analysis

The GFPT2 expression, the expression levels of JAK STAT signaling pathway-related genes (JAK1, JAK2, JAK2, TYK2, STAT1, STAT2, STAT3, STAT4, STAT5A, STAT5B, STAT6) were obtained from COAD samples in TCGA dataset. The heatmap was visualized with ggplot2. Pearson correlation coefficient tests were used to estimate the association between the GFPT2 expression and other genes.

### Corrplot of GFPT2 and Other Factors

The GFPT2 expression and other factors (CAFs-associated factors, EMT-related factors, T cell exhaustion factors, immunosuppressive factors) were obtained from COAD samples in TCGA dataset. The corrplot package was performed and the pearson correlation coefficient tests were used to estimate the association between the GFPT2 expression and other factors.

### Drug Sensitivity Analysis

Gene expression and drug sensitivity data were downloaded from CellMiner dataset, and we removed drugs without clinical trials or FDA approval. The correlation coefficients between GFPT2 expression and drug sensitivity were calculated using the cor.test function in R language, and correlation tests were done. We defined P < 0.05 as the correlation between the target gene and drug sensitivity was significant. If the correlation coefficient was greater than 0, it means that there was a positive correlation between GFPT2 expression and drug sensitivity.

## Results

### GFPT2 Is Highly Expressed in Colon Cancer and Correlates With Poor Pathological Features

**Figure 1:**
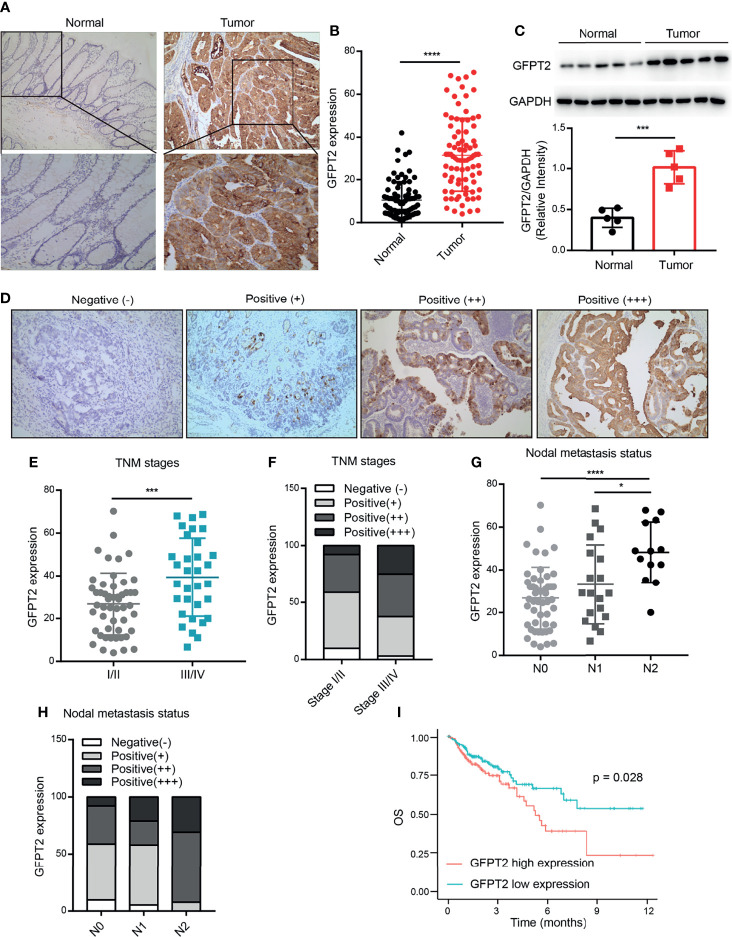
GFPT2 is highly expressed in colon cancer and correlates with poor pathological features. **(A)** The typical images of GFPT2 expression levels in colon cancer tissues and corresponding paracancerous tissues by immunohistochemistry (IHC). **(B)** GFPT2 was shown to be significantly higher in colon cancer tissues compared to paracancerous tissues. **(D)** Representative images of different GFPT2 (negative-, positive+, positive++, positive ++++) protein levels by IHC. **(C)** Western blot analysis of GFPT2 expression levels in 5 pairs of colon cancer tissues and paracancerous tissues; GAPDH was used as the loading control. **(E)** GFPT2 protein levels from different TNM stages (I/II stage or III/IV stage) in 83 colon cancer tissues. **(F)** The different GFPT2 positive levels (-, +, ++, ++++) in I/II stages were 9.80%, 49.02%, 33.33% and 7.85%, respectively; and 3.12%, 34.38%, 37.50% and 25.00% in III/IV stage, respectively. **(G)** GFPT2 protein levels from different NODE metastasis status (N0, N1, N2) in 83 colon cancer tissues. **(H)** The positive levels of different GFPT2 (-, +, ++, ++++) were 9.80%, 49.02%, 33.33% and 7.85% in N0 stage, respectively; and 5.26%, 52.63%, 21.05% and 21.06% in N1 stage, respectively; and 0.00%, 7.69%, 61.54% and 30.77% in N2 stage, respectively. **(I)** The overall survival (OS) of GFPT2 in the TCGA database was used by Kaplan-Meier in 329 COAD samples from TCGA dataset. *P < 0.05; ***P < 0.001; ****P < 0.0001.

To explore the roles of GFPT2 in patients with colon cancer, we first investigated the expression levels of GFPT2 in colon cancer. We collected tissues from 83 colon cancer patients and detected the protein levels of GFPT2 by immunohistochemistry (IHC) assy. **Figure 1A** shows typical images of GFPT2 expression levels in colon cancer tissues and corresponding paraneoplastic tissues. Our results showed that GFPT2 was an oncogene highly expressed in colon cancer tissues compared to paraneoplastic tissues (**Figures 1A, B**). Western Blot analysis also further confirmed that GFPT2 protein levels were significantly higher in colon cancer tissues than in paracancerous tissues (**Figure 1C**).

We further investigated the correlation between the expression levels of GFPT2 and the pathological characteristics of colon cancer patients. As shown in **Table 1**, high levels of protein expression of GFPT2 were positively correlated with unfavorable clinicopathological features in 83 samples of colon cancer patients. Specifically, increased GFPT2 levels were positively associated with advanced TNM (χ^2^ = 7.803, P=0.005 <0.01), advanced primary tumor size (χ^2^ = 5.896, P=0.015 <0.05) and excessive lymph node metastasis (χ^2^ = 10.008, P=0.007 <0.01). However, protein expression of GFPT2 showed no correlation in patient’s gender, age, and cell differentiation (P > 0.05). We showed the representative images of the protein levels of different GFPT2 (negative-, positive+, positive++, positive ++++) in **Figure 1D**. Our results showed that more advanced TNM stages had higher GFPT2 expression levels than early TNM stages (**Figure 1E**). Moreover, the different GFPT2 positive levels (-, +, ++, ++++) in early TNM stages were 9.80%, 49.02%, 33.33% and 7.85%, respectively; and 3.12%, 34.38%, 37.50% and 25.00% in late TNM, respectively (**Figure 1F**). In addition, more cases of lymph node metastasis were positively correlated with higher GFPT2 levels (**Figure 1G**). The positive levels of different GFPT2 (-, +, ++, ++++) were 9.80%, 49.02%, 33.33% and 7.85% in N0 stage, respectively; and 5.26%, 52.63%, 21.05% and 21.06% in N1 stage, respectively; and 0.00%, 7.69%, 61.54% and 30.77% in N2 stage, respectively (**Figure 1H**).

**Table 1:** Association between GFPT2 protein expression and clinicopathological characteristics in colon cancer tissues.

Clinicopathological characteristic	n	GFPT2 expression		χ^2^	*P* value
		Low expression	High expression		
**Total number**	83	42	41		
**Gender**				0.003	0.958
** Male**	49	23 (46.94)	26 (53.06)		
** Female**	34	19 (55.88)	15 (44.12)		
**Age (years)**				0.071	0.790
** <65**	38	21 (55.26)	17 (44.74)		
** ≥65**	45	21 (46.67)	24 (53.33)		
**Cell differentiation**				2.049	0.359
** Well**	21	9 (42.86)	12 (57.14)		
** Moderate**	36	19 (52.78)	17 (47.22)		
** Poor**	26	9 (34.62)	17 (65.38)		
**Primary tumor size**				5.896	0.015*
** T1-2**	48	30 (62.50)	18 (37.50)		
** T3-4**	35	12 (34.29)	23 (65.71)		
**Lymph node metastasis**				10.008	0.007^**^
** N0**	51	32 (62.75)	19 (37.25)		
** N1**	19	8 (42.11)	11 (57.89)		
** N2**	13	2 (15.38)	11 (84.62)		
**TNM**				7.803	0.005^**^
** I/II**	51	32 (62.75)	19 (37.25)		
** III/IV**	32	10 (31.25)	22 (68.75)		

^**^P < 0.01.

To further explore the relationship between GFPT2 expression and prognosis of colon cancer patients, we investigated the overall survival (OS) of GFPT2 in the TCGA database using Kaplan-Meier. The results showed that high GFPT2 expression was significantly associated with poorer OS in 329 patients with colon adenocarcinoma (COAD) (P=0.028 < 0.05) (**Figure 1I**). We concluded that GFPT2 expression levels were associated with poor pathological characteristics and poor prognostic features in patients with colon cancer.

### GFPT2 Is Associated With Tumor-Associated Enrichment Pathways

**Figure 2:**
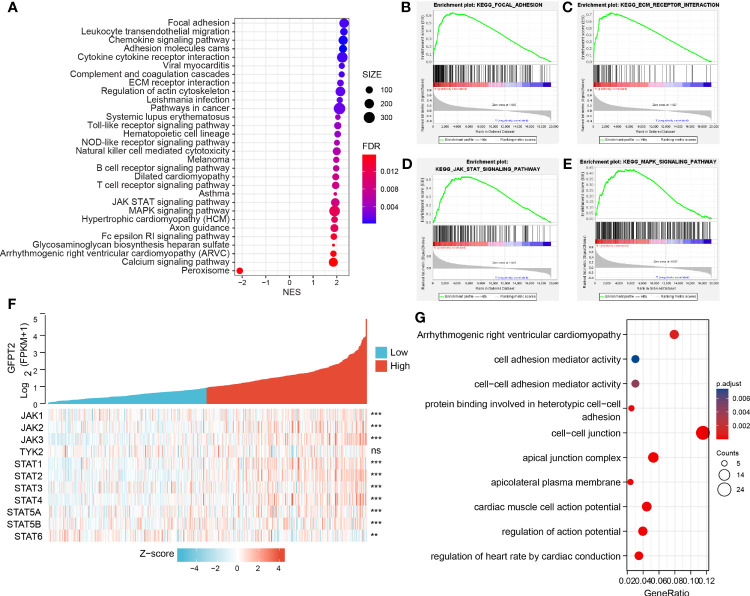
GFPT2 is associated with tumor-associated enrichment pathways. **(A–E)** KEGG enrichment pathways were performed using the clusterProfilter R package. **(F)** Heat map showing the relationship between GFPT2 and JAK-STAT signaling pathway-related genes. **(G)** GO and KEEG analysis were used to check the related functions and signaling pathways of GFPT2 in the sequencing results of CRC cell lines from CCLE database. **P < 0.01; ***P < 0.001; ns, no significance.

To explore the expression and pathway enrichment of GFPT2 in the TCGA database, we analyzed GFPT2 and related genes in COAD cases. We performed GO analysis using the ClusterProfiler R package and obtained GFPT2 significantly enriched functions and pathways (P < 0.05). We displayed 30 pathways significantly enriched with GFPT2 that were associated with important tumor-related pathways, including pathways in cancer, Focal adhesion, Adhersion molecules cams, ECM receptor interaction, JAK-STAT signaling pathway and MAPK signaling pathway (**Figures 2A–E**), suggesting that GFPT2 may play important function in tumorigenesis and progression of colon cancer. In cell biology, focal adhesion and ECM receptor interaction mediate the regulation of cell adhesion to the extracellular matrix (ECM) mainly through its transmission between the ECM and interacting cells (18, 19). They, therefore, play a central role in cell migration (19). Increased GFPT2 levels were positively linked to the enrichment of focal adhesion and ECM receptor interaction (**Figures 2B, C**), indicating that GFPT2 may have the essential position in stromal cell and invasive of tumors. Similarly, in the CCLE database, GFPT2 correlation analysis showed similar pathways as TCGA, such as cell adhesion mediator activity, protein binding involved in heterotypic cell-cell adhesion, cell-cell junction (**Figure 2G**).

The JAK-STAT signaling pathway is an important pathway in cell biology that is involved in various vital physiological functions, including immunity, cell death and tumor formation (20). Abnormalities in JAK-STAT signaling will lead to various diseases, such as skin diseases, cancer and diseases affecting the immune system (21). It has four JAK proteins, namely JAK1, JAK2, JAK3 and TYK2 (21). Seven STAT proteins are involved, which can be identified as STAT1, STAT2, STAT3, STAT4, STAT5A, STAT5B and STAT6 (21). We demonstrated a remarkable positively related correlation between GFPT2 overexpression and proteins associated with the JAK-STAT signaling pathway (**Figure 2F**). JAK-STAT signaling is able to interconnect with other cellular signaling pathways, such as the MAPK/ERK pathway. JAKs phosphorylated receptors can bind to the SH2 binding domain of Grb2, an important protein in the MAPK/ERK pathway (20). In addition, MAPK (mitogen-activated protein kinase), can phosphorylate STATs, thus allowing STATs to increase gene transcription, which in turn promotes the JAK-STAT signaling pathway (20). Interestingly, our results revealed that an increase in GFPT2 promotes the MAPK signaling pathway (**Figure 2E**), a result consistent with previous published reports.

Interestingly, in addition to GFPT2 high expression enriching lots of tumor-related pathways, increased GFPT2 expression also enriched a large number of immune-related pathways, including Chemokine signaling pathway, Cytokine cytokine receptor interaction, Toll-like receptor signaling pathway, NOD-like receptor signaling pathway, Natural killer cell mediated cytotoxicity, B cell receptor signaling pathway and T cell receptor signaling pathway (**Figure 2A**).

### Association of GFPT2 Levels With Tumor Microenvironment

**Figure 3:**
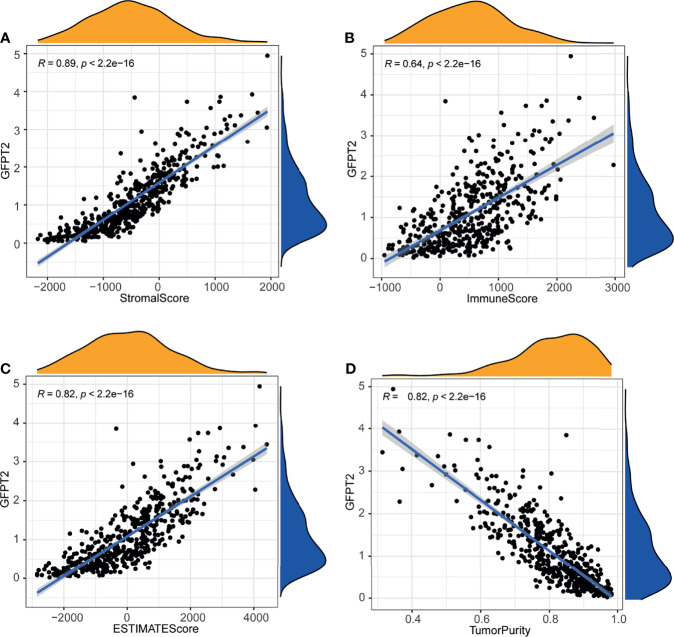
Association of GFPT2 levels with tumor microenvironment. The association between GFPT2 expression levels and stromal score **(A)**, immune score **(B)**, and ESTIMATE score **(C)** and tumor purity score **(D)**.

We have known that GFPT2 expression related to ECM and immune pathways, since stromal cells and immune cells are major components of the tumor microenvironment (TME), so we speculated whether GFBP2 would be involved in TME. To confirm our hypothesis, we observed the correlation of GFPT2 expression with stromal cell and immune cell infiltrations. Our results revealed that increased GFPT2 expression was strongly and positively associated with stromal score (R=0.89, P<0.0001) (**Figure 3A**), immune score (R=0.64, P<0.0001), and ESTIMATE score (a combined score of stromal and immune cells) (R=0.82, P<0.0001) (**Figures 3A–C**), indicating that GFPT2 expression affects stromal and immune cell infiltrations. Interestingly, the high levels of GFPT2 were significantly and negatively linked to tumor purity (R=0.82, P<0.0001) (**Figure 3D**), suggesting that GFPT2 mainly affects the predominant activity of TME, which has essential position in stromal cell and immune cell infiltrations.

### Correlation Between GFPT2 Levels and Stromal Cell Infiltration

**Figure 4:**
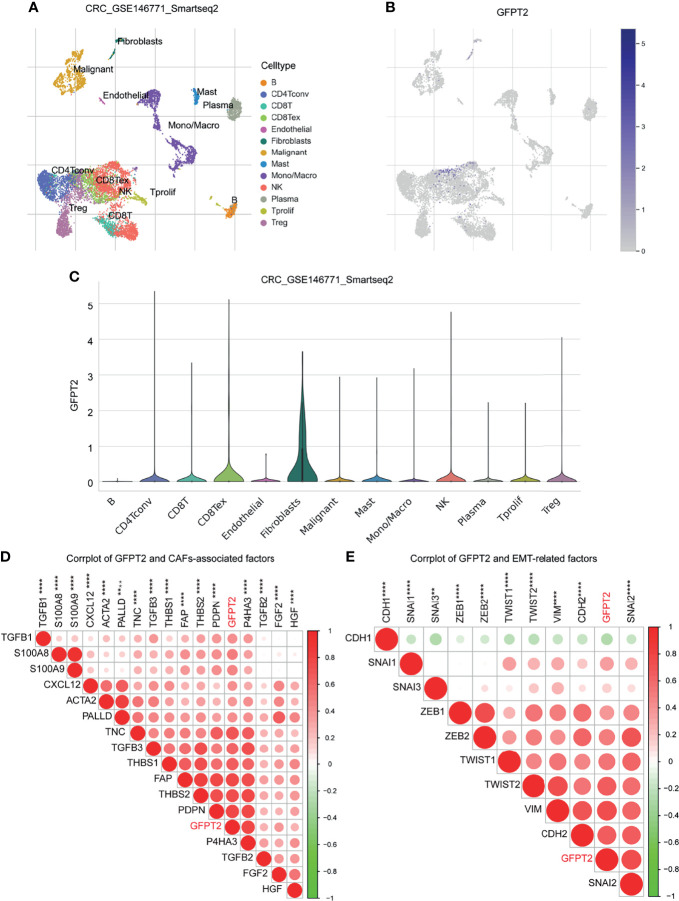
Correlation between GFPT2 levels and stromal cell infiltration. **(A–E)** GFPT2 was expressed in both immune and stromal cell single cell subpopulations in a colon cancer single cell GSE dataset (GSC_GSE146771_Smartseq2) from TISCH dataset. **(D)** Corrplot was used to perform the correlation between GFPT2 levels and CAFs-associated factors. **(E)** Corrplot was used to perform the correlation between GFPT2 levels and EMT-related factors. **P < 0.01; ****P < 0.0001.

The TME is composed of ECM, cancer-associated fibroblasts (CAFs), myofibroblasts, immune cells and other factors (22). In order to examine the relationship between GFPT2 levels and stromal cell infiltrations, we first surveyed the Tumor Immune Single-cell Hub (TISCH) (http://tisch.comp-genomics.org/) database (a single cell center) to investigate which cell subpopulations of GFPT2 are primarily expressed in. We explored a colon cancer single cell GSE dataset (GSC_GSE146771_Smartseq2) and found that GFPT2 was expressed in both immune and stromal cell single cell subpopulations (**Figures 4A–C**). Since the tumor-associated stromal cells mainly include endothelial cells, fibroblasts and myofibroblasts. We next investigated which stromal cell components were the cell subpopulations with high GFPT2 expression in colon cancer. The findings indicated that fibroblasts were the major GFPT2 expressing cells, and the expression of GFPT2 was very high in fibroblasts compared to other cell subpopulations (**Figure 4C**), indicating that GFPT2 has an important function in CAFs.

Next, we inspected the association among GFPT2 mRNA expression and biomarkers associated with CAFs. CAFs primarily express α-smooth muscle actin (α-SMA), fibronectin (FAP), cytoskeletal protein (Palladin), mucin-type protein (podoplanin), and prolyl 4-hydroxylase, while TGF-β, PDGF, HGF, FGF2 and THBS1 are the main factors that promote the activation of CAFs (23–25). S100A8/A9 could facilitate the proliferation of fibroblasts and worked in the differentiation of fibroblasts to myofibroblasts (26). In the current study, we noticed that the expression of GFPT2 had significantly positive correlation with the above mentioned markers related to CAFs (**Figure 4D**).

CAFs exert huge contributions in tumorigenesis and development, which can mainly boost angiogenesis, promote the initiation of EMT and affect the survival of tumor cells (27). To confirm the effects of GFPT2 expression on EMT, we examined the levels of GFPT2 in relation to EMT-related markers. The results showed that GFPT2 expression was highly correlated with EMT-related factors (**Figure 4E**).

### Association of GFPT2 Levels With Immune Cell Infiltration

**Figure 5:**
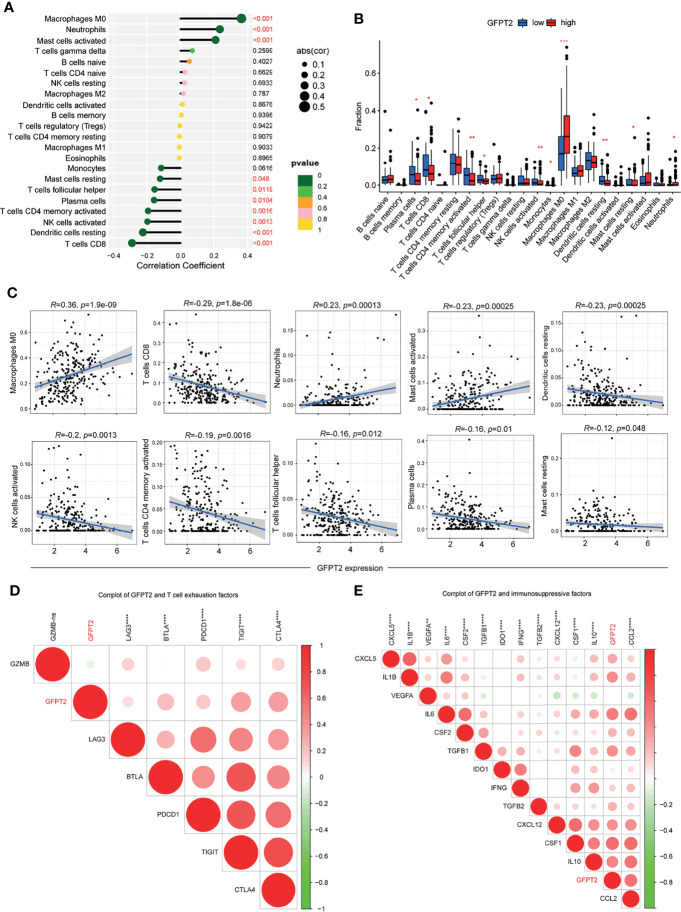
Association of GFPT2 levels with immune cell infiltration. **(A–C)** The correlation coefficients between GFPT2 expression and immune cells using the CIBERSORT method. **(D)** Corrplot was used to perform the correlation between GFPT2 levels and T cell exhaustion factors. **(E)** Corrplot was used to perform the correlation between GFPT2 levels and immunosuppressive factors. *P < 0.05; **P < 0.01; ***P < 0.001; ****P < 0.0001.

To better evaluate the influences of GFPT2 levels on immune cell infiltration, we calculated the correlation coefficients between GFPT2 expression and immune cells using the CIBERSORT method. The correlation between GFPT2 expression and immune cells was verified by spearman’s correlation test. There was a statistically significant positive correlation between increasing GFPT2 and immune cell fractions, including Macrophages M0, Neutrophils and activated Mast cells (**Figures 5A–C**). However, overexpression of GFPT2 adversely related to certain immune cell components, such as Monocytes, resting Mast cells, T cells follicular helper, plasma cells, activated CD4 T cells memory, activated NK cells, resting Dendritic cells, CD8 T cells (**Figures 5A–C**).

In general, T-cell depletion is vital factor for the effectiveness of immune checkpoint blockade (28). To further discuss the relevance of GFPT2 levels to marker genes of T-cell exhaustion, our results displayed a remarkable association of GFPT2 levels with LAG3, BTLA, PDCD1, TIGIT and CTLA4 (**Figure 5D**). Our analysis suggested that GFPT2 was mainly positively correlated with immunosuppressive cells, such as fibroblasts and macrophages, and therefore, we hypothesized that GFPT2 might be involved in the regulation of COAD as an immunosuppressive component. To verify this point, we conducted a correlation analysis between GFPT2 expression and key factors of immunosuppression. The results showed that GFPT2 levels were significantly and positively correlated with many immunosuppressive factors (**Figure 5E**).

### The Relationship Between GFPT2 Expression Levels and Drug Sensitivity

**Figure 6:**
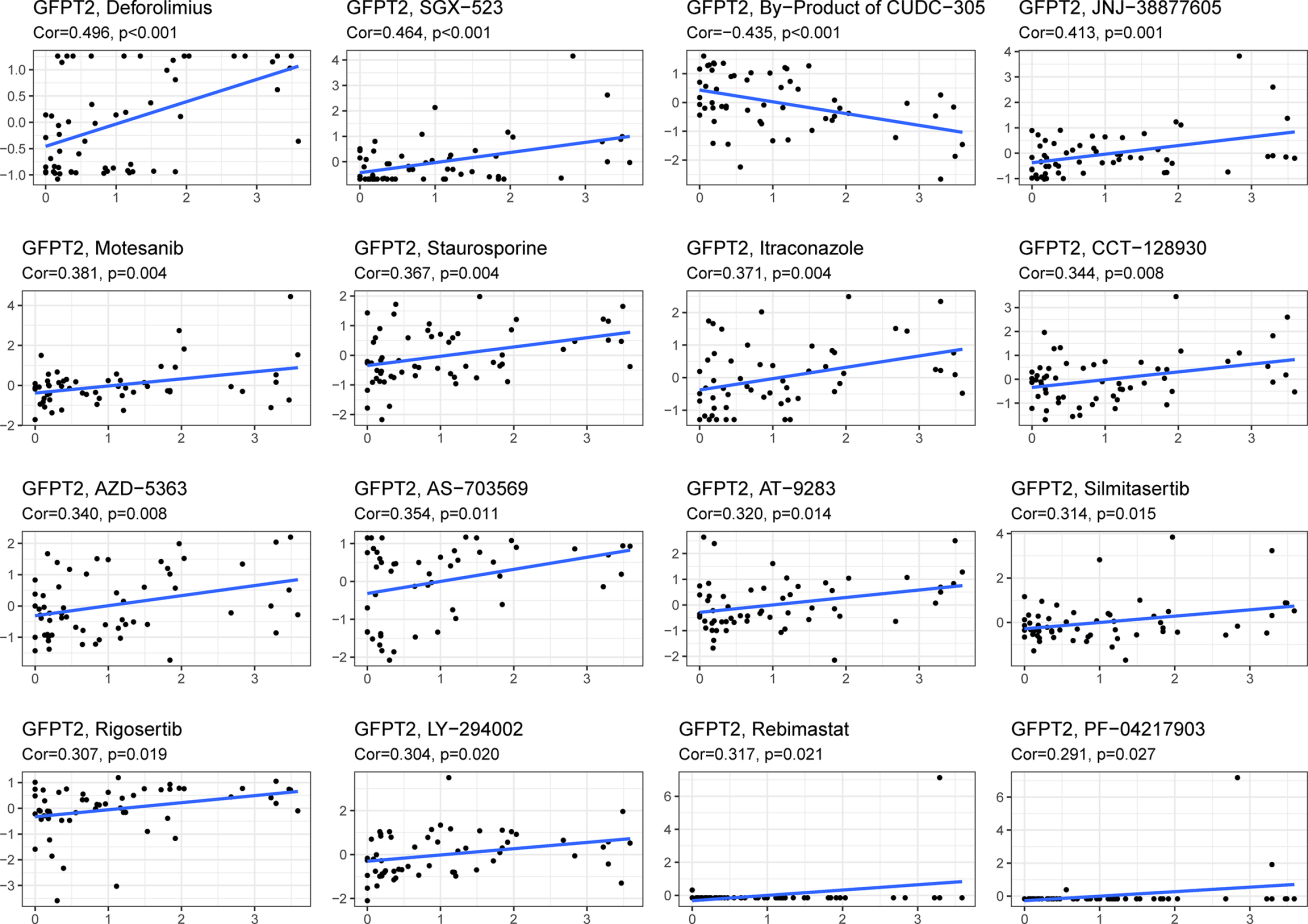
The relationship between GFPT2 expression levels and drug sensitivity. The correlation coefficient between GFPT2 expression and drug sensitivity from CellMiner dataset.

We downloaded gene expression and drug sensitivity data from CellMiner and removed drugs without clinical trials or FDA approval and calculated the correlation coefficient between GFPT2 expression and drug sensitivity using the cor.test function and correlation in R language. We selected the top 16 drugs associated with GFPT2 by R value, and the results showed that GFPT2 expression was associated with a number of drugs, including Deforolimius, SGX-523, JNJ-38877605, Motesanib, Staurosporine, Itraconazole, CCT-128930, AZD-5363, AS-703569, AT-9283, Silmitasertib, Rigosertib, LY-294002, Rebimastat, PF-04217903 (**Figure 6**), and the higher GFPT2 expression was linked to better sensitivity of tumor cells to these drugs. And the expression level of GFPT2 was associated with increased resistance to By-Product of CUDC-305 (**Figure 6**). These results suggest that the expression of GFPT2 may be a judgment of the sensitivity of a certain class of drugs.

## Discussion

GFPT2, the rate-limiting enzyme of HBP, plays an important role in the metabolic activity of cells, especially in their glucose metabolism (29). Recently Liu et al. demonstrated that GFPT2 promoted metastasis and formed a positive feedback loop with p65 in colorectal cancer (29). Moreover, it was also reported that elevated GFPT2 expression was correlated with poor clinical outcome in non-small cell lung cancer (30). In our study, we found that GFPT2 was aberrantly expressed in CRC tumor tissues relative to normal tissues and that GFPT2 expression and clinical TNM staging as well were positively correlated, and analysis of the TCGA database also revealed that patients with high GFPT2 expression had decreased survival time. These results suggest that GFPT2 plays a facilitating role in the progression of colon cancer.

By analyzing the KEGG and GO signaling pathways, we found that GFPT2 affects the development of colon cancer mainly through Focal adhesion, and ECM receptor interaction. Both of these are related to cell migration and interactions between receptors of extracellular mechanisms (31, 32). Focal adhesion kinases (FAKs) are a class of cytoplasmic non-receptor protein tyrosine kinases that belong to the protein tyrosine kinase (PTK) superfamily and are therefore also known as PTK II (32). FAKs can integrate signals from integrins, growth factors and mechanical stimuli to activate intracellular PI3K/Akt, Ras/MAPK and other signaling pathways to regulate cell growth (33–35). Numerous studies on the association of FAKs with different types of cancers have shown a close link between FAKs and the biological mechanisms that promote cancer development and progression (36). Moreover, FAKs tend to be inversely associated with better clinical cancer sample outcomes, and related studies have found that FAKs are overexpressed and/or hyperphosphorylated in a variety of cancer cells and are responsible for cell migration, survival, proliferation and adhesion (37, 38). Recently studys found that FAKs promote renewal and drug resistance in cancer stem cells (CSCs) by acting in survival signaling (39). For example, FAKs and extracellular signal-regulated kinase (ERK1/2) pathways are involved in regulating the growth and metastasis of liver cancer stem cells (40).

ECM is an essential component of stromal cells, therefore, we hypothesized that GFPT2 might have regulatory effects on TME, since stromal cells were a critical component of TME (41). Cancer development and progression often coincide with changes in the surrounding stroma (42). Cancer cells can functionally shape the microenvironment by secreting different chemokines and chemotactic factors (43). Once the microenvironment is formed, this leads to a reprogramming of the surrounding cells, which enables them to play a crucial function in the survival and progression of the tumors (44). Interestingly, in the current study, increased GFPT2 expression was highly associated with stromal cells, especially CAFs, we also found that the expression of GFPT2 had significantly positive correlation with CAFs-related factors.

During cancer progression, epithelial tumors may undergo EMT, which alters the characteristics of tumor cells, resulting in the loss of the epithelial marker E-calcineurin and an increase in the mesenchymal markers N-calcineurin, fibronectin, and wave proteins (45, 46). Numerous reports have shown that CAFs and tumor-associated macrophages (TAMs) can contribute to cancer cell adhesion and growth, playing critical roles in ECM alterations (47). This process enhances the separation of cancer cells from the primary tumors and enhances invasiveness, thus allowing cancer cells to enter the bloodstream or lymph and eventually lead to distant metastasis (48). Here, GFPT2 expression was highly correlated with EMT-related factors, indicating that GFPT2 might be capable of regulating the promotion of colon cancer metastasis. This speculation could be confirmed to certain extent by the high expression of GFPT2 with more lymph node metastasis. However, we will perform some cellular and animal experiments in the future to further confirm whether GFPT2 is associated with colon cancer.

Immune cells are another important component of TME, and a growing number of evidences suggest that the presence of innate immune cells (macrophages, neutrophils, dendritic cells, and natural killer cells, etc.) as well as adaptive immune cells (T cells and B cells) in TME promoter tumor progression (42). In this study, increasing GFPT2 expression was significantly associated with immune cells, and positively correlated with immunosuppressive cells and T-cell exhaustion. Therefore, GFPT2 may be involved in tumor cell immune escape. Currently, immunotherapies targeting immune checkpoints, such as anti-PD-L1 antibodies, have shown clinical activity against various types of cancer, and the increase in immune checkpoints can suppress the anti-tumor immune response of T cells (49). Inhibition of the antitumor immune response of T cells by increasing the expression of PD-1 and CTLA4 receptors makes GFPT2 a potential new target for immunotherapy.

However, how does GFPT2 modulate the role of immune cells? The JAK-STAT signaling pathway mediates numerous tumor immunomodulatory processes, including tumor cell recognition and tumor immune escape (50). The anti-tumor immune response is dominated by two of these factors, STAT1 and STAT2, which act by inducing type I and type II interferons (IFN) (51). Conversely, STAT3 mainly maintains cancer cells survival and regulates immunosuppression and continuous inflammation in TME (52). In this study, we demonstrated a remarkable positively related correlation between GFPT2 overexpression and proteins associated with the JAK-STAT signaling pathway. Therefore, we speculate that GFPT2 may mediate the regulation of immunosuppression mainly through the JAK STAT signaling pathway. In the future, we will design some animal experiments to confirm our speculations.

Tolwani et al. suggested that the strong expression of GFPT2 in primary leukodystrophies may be associated with high metabolic activity, and the expression of GFPTs may be also implicated in the reprogramming of the TME (53). Furthermore, overexpression of GFPTs in hepatocellular carcinoma cells was also reported to increase transcript levels of lipogenic genes (54). Indeed, metabolic reprogramming of the TME is recognized as a cancer hallmark. Genes implicated in hexosamine biosynthetic pathway including GFPT were demonstrated to be promoted in hypoxic tumor cells (55). Numerous studies have shown that hypoxia can induce modifications in cancer cell metabolism (56, 57). Furthermore, hypoxia can instigate pronounced remodeling of the CAF proteome (58). Components of the TME including CAFs in turn are known to play pivotal supportive roles in tumor growth and progression and CAFs are in a reciprocal communication with the tumor cells in the tumor milieu (59). In a seminal study, Zhang et al. reported that normal fibroblasts transformed to CAF-like cells, following TGF-β treatment, upregulated hexosamine biosynthesis pathway genes, including GFPT2 (60). Such previous findings would indeed agree with the current finding of association between GFPT2 expression and prognosis as well as TME (e.g. CAFs) of colon cancer.

## Conclusions

In this study, we found that GFPT2 and colon cancer are closely related, and we identified the value of GFPT2 in patient prognosis prediction, tumor microenvironment, tumor immunity and drug sensitivity through the analysis of TCGA database and validation of clinical samples. Currently, the specific underlying mechanism between GFPT2 expression and tumor immunity in colon cancer remains unclear, which deserves further investigation. Overall, our work largely revealed the roles of GFPT2 in tumorigenesis, especially in immune response, tumor microenvironment and drug resistance, which is crucial for the development of customized cancer therapies.

## Data Availability Statement

The original contributions presented in the study are included in the article/supplementary material. Further inquiries can be directed to the corresponding author.

## Ethics Statement

The studies involving human participants were reviewed and approved by the committee of the Affiliated Huaian No.1 People’s Hospital of Nanjing Medical University. The patients/participants provided their written informed consent to participate in this study.

## Author Contributions

XD and XZ designed the study. XD and HL performed the IHC experiment. YY and QZ analyzed the data. XD, HL, and XZ discussed the project. XD and HL drafted the manuscript. XZ proofread and revised the manuscript. All authors contributed to the article and approved the submitted version.

## Conflict of Interest

The authors declare that the research was conducted in the absence of any commercial or financial relationships that could be construed as a potential conflict of interest.

## Publisher’s Note

All claims expressed in this article are solely those of the authors and do not necessarily represent those of their affiliated organizations, or those of the publisher, the editors and the reviewers. Any product that may be evaluated in this article, or claim that may be made by its manufacturer, is not guaranteed or endorsed by the publisher.

## Abbreviations

GFPT2, Glutamine-fructose-6-phosphate transaminase 2; IHC, immunohistochemistry; OS: overall survival; TCGA, The Cancer Gene Atlas; TISCH, The Tumor Immune Single Cell Center; COAD, colon adenocarcinoma; GO, Gene ontology; KEGG, Kyoto Encyclopedia of Genes and Genomes; TME, tumor microenvironment; TCA, tricarboxylic acid; HBP, hexosamine biosynthetic pathway; FPKM, Fregments Per Kilobase per Million; ESTIMATE, Estimation of STromal and Immune in MAlignant Tumors Using Expression; ECM, extracellular matrix; CAFs, cancer-associated fibroblasts; α-SMA, α-smooth muscle actin; FAP, fibronectin; EMT, epithelial-mesenchymal transition; FAKs, Focal adhesion kinases; PTK, protein tyrosine kinase; CSCs, cancer stem cells; IFN, interferons.
